# Supplementary figures and images for: Impact of vented and condenser tumble dryers on waterborne and airborne microfiber pollution
Source: PLoS One. 2023 May 24;18(5):e0285548. doi: 10.1371/journal.pone.0285548 (PMC10208492; doi:10.1371/journal.pone.0285548)

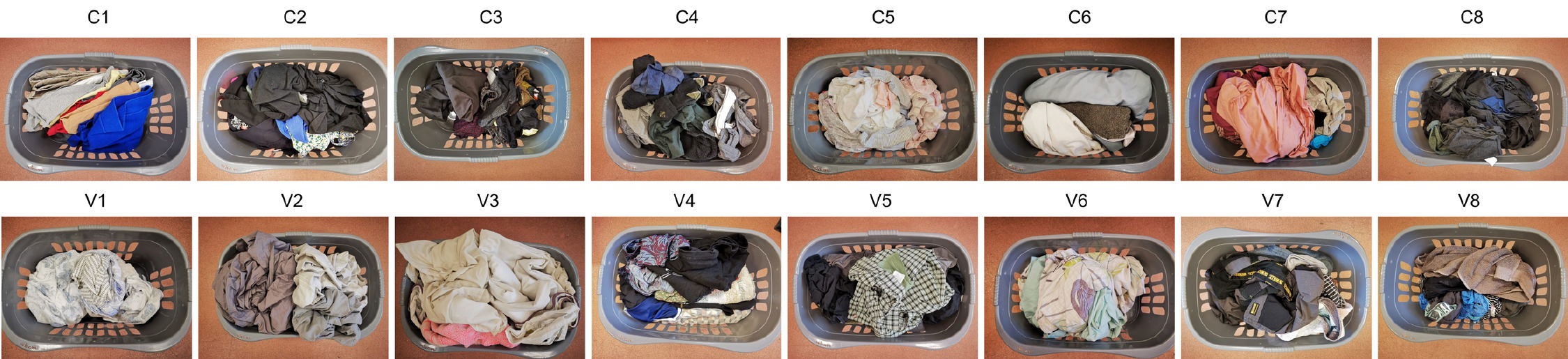

Supplement: S1 Fig — Photos C1-C8 and V1-V8 show the soiled consumer loads used for the testing with a condenser dryer (C) and vented dryer (V). (TIF) [file pone.0285548.s001.tif]

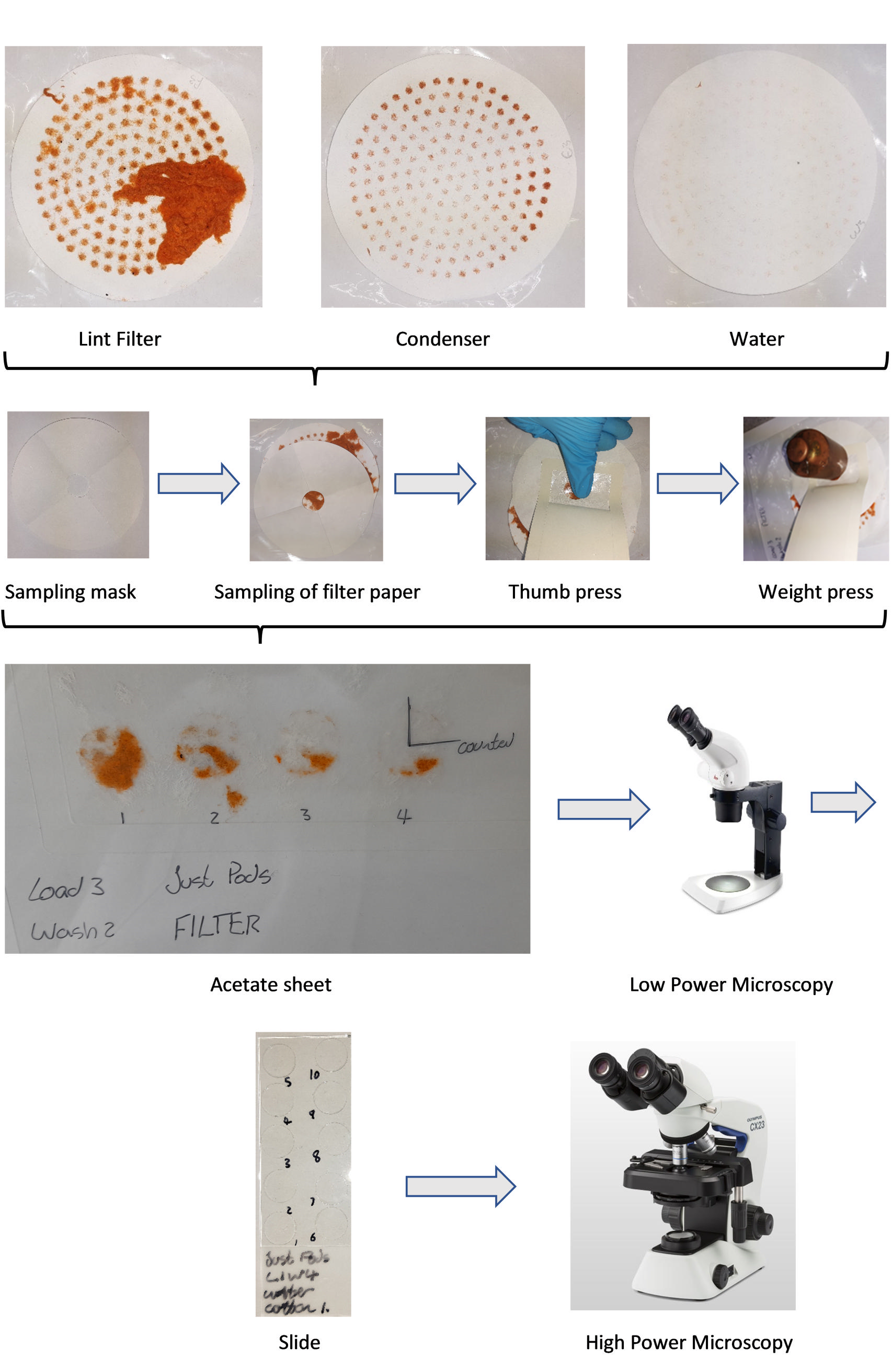

Supplement: S2 Fig — Microfibers filtered from each point of collection are recovered using adhesive tape and transferred to acetate sheet for determination of fiber composition and dimensions by microscopy. (TIF) [file pone.0285548.s002.tif]
