# Supplementary material for: Impact of vented and condenser tumble dryers on waterborne and airborne microfiber pollution
Source: PLoS One. 2023 May 24;18(5):e0285548. doi: 10.1371/journal.pone.0285548 (PMC10208492; doi:10.1371/journal.pone.0285548)
Supplement: S1 Table — The table shows measured mass of the wash load used (kg) and microfibers collected (mg) on the dryer lint filter, on the condenser and in the condensed water. These data are used to calculate quantity of microfibers at these three stages in terms of ppm (parts per million, i.e., mg microfiber released per kg dry wash load) and percentage lint filter efficiency for each of the four drying cycles. (DOCX) [file pone.0285548.s003.docx]

**S1** **Table. Gravimetric quantification of microfiber release from clean T-shirts in condenser tumble dryers.** The table shows measured mass of the wash load used (kg) and microfibers collected (mg) on the dryer lint filter, on the condenser and in the condensed water. These data are used to calculate quantity of microfibers at these three stages in terms of ppm (parts per million, i.e. mg microfiber released per kg dry wash load) and percentage lint filter efficiency for each of the four drying cycles.

| **Cycle** | **Load** | **Load mass (kg)** | **Microfiber mass** | | | **Microfiber release^*^** | | | | **Lint filter efficiency^†^** | | **Reduction in microfiber release**  **%** | | | |
| --- | --- | --- | --- | --- | --- | --- | --- | --- | --- | --- | --- | --- | --- | --- | --- |
|  |  |  | **(mg)** | | | **(ppm)** | | | | % | |  |  |  |  |
|  |  |  | **Lint Filter** | **Condenser** | **Water** | **Lint Filter** | **Condenser** | **Water** |  | | **Cycle link** | | **Lint Filter** | **Condenser** | **Water** |
| 1 | 1 | 2.84 | 868.5165 | 37.2524 | 11.7065 | 305.8157 | 13.1171 | 4.1220 | 94.7 | |  |  |  |  |  |
|  | 2 | 2.84 | 816.6500 | 50.6318 | 11.4611 | 287.5528 | 17.8281 | 4.0356 | 92.9 | |  |  |  |  |  |
|  | 3 | 2.83 | 870.1307 | 51.1916 | 17.2992 | 307.4667 | 18.0889 | 6.1128 | 92.7 | | **1 to 2** | | -34.5 | -14.8 | +19.2 |
|  | **Mean** | **2.84** | **851.7657** | **46.3586** | **13.4889** | **300.2784** | **16.3447** | **4.7568** | **93.4** | |  |  |  |  |  |
|  | **Std Dev** | **0.01** | **30.4219** | **7.8912** | **3.3021** | **11.0516** | **2.7983** | **1.1751** | **1.1** | |  |  |  |  |  |
| 2 | 1 | 2.84 | 531.2198 | 32.7213 | 10.4992 | 187.0492 | 11.5216 | 3.6969 | 92.5 | |  |  |  |  |  |
|  | 2 | 2.84 | 611.3210 | 46.4130 | 16.3231 | 215.2539 | 16.3426 | 5.7476 | 90.7 | |  |  |  |  |  |
|  | 3 | 2.83 | 531.6561 | 39.3455 | 21.3340 | 187.8643 | 13.9030 | 7.5385 | 89.8 | | **2 to 3** | | -35.1 | -40.1 | -21.7 |
|  | **Mean** | **2.84** | **558.0656** | **39.4933** | **16.0521** | **196.7225** | **13.9224** | **5.6610** | **91.0** | |  |  |  |  |  |
|  | **Std Dev** | **0.01** | **46.1210** | **6.8471** | **5.4225** | **16.0538** | **2.4106** | **1.9223** | **1.4** | |  |  |  |  |  |
| 3 | 1 | 2.84 | 373.1605 | 14.8775 | 16.8231 | 131.3945 | 5.2385 | 5.9236 | 92.2 | |  |  |  |  |  |
|  | 2 | 2.84 | 358.1826 | 30.6775 | 10.4333 | 126.1206 | 10.8019 | 3.6737 | 89.7 | |  |  |  |  |  |
|  | 3 | 2.83 | 354.4658 | 25.4119 | 10.4789 | 125.2529 | 8.9795 | 3.7028 | 90.8 | | **3 to 4** | | -20.8 | -26.1 | -42.2 |
|  | **Mean** | **2.84** | **361.9363** | **23.6556** | **12.5785** | **127.5894** | **8.3400** | **4.4334** | **90.9** | |  |  |  |  |  |
|  | **Std Dev** | **0.01** | **9.8965** | **8.0451** | **3.6761** | **3.3238** | **2.8363** | **1.2907** | **1.2** | |  |  |  |  |  |
| 4 | 1 | 2.84 | 297.9706 | 17.7481 | 7.8340 | 104.9192 | 6.2493 | 2.7584 | 92.1 | |  |  |  |  |  |
|  | 2 | 2.84 | 266.6579 | 16.7347 | 8.2213 | 93.8936 | 5.8925 | 2.8948 | 91.4 | |  |  |  |  |  |
|  | 3 | 2.83 | 295.7391 | 17.9261 | 5.7565 | 104.5014 | 6.3343 | 2.0341 | 92.6 | |  | | | | |
|  | **Mean** | **2.84** | **286.7892** | **17.4696** | **7.2706** | **101.1048** | **6.1587** | **2.5625** | **92.0** | |  |  |  |  |  |
|  | **Std Dev** | **0.01** | **17.4699** | **0.6427** | **1.3255** | **6.2485** | **0.2344** | **0.4626** | **0.6** | |  |  |  |  |  |
| All | **Mean** |  |  |  |  | **181.4237** | **11.1914** | **4.3534** | **91.8** | |  |  |  |  |  |
|  | **Std Dev** |  |  |  |  | **80.8979** | **4.7281** | **1.6285** | **1.4** | |  |  |  |  |  |

***Microfiber release (ppm) = Microfiber mass (mg) / Load mass (kg)**

**^†^Lint filter efficiency is the percentage of total microfiber release collected on the lint filter**
