# Supplementary material for: Impact of vented and condenser tumble dryers on waterborne and airborne microfiber pollution
Source: PLoS One. 2023 May 24;18(5):e0285548. doi: 10.1371/journal.pone.0285548 (PMC10208492; doi:10.1371/journal.pone.0285548)
Supplement: S2 Table — The table shows the analyzed fiber composition of the microfiber samples collected on the lint filter, condenser and in the condensed water. Columns ‘Cotton’ and ‘Polyester’ contain the number of fibers of each type identified within the sample. These are used to calculate the percentage composition. (DOCX) [file pone.0285548.s004.docx]

**S2 Table. Fiber composition of microfibers released from clean T-shirts in condenser tumble dryers.** The table shows the analyzed fiber composition of the microfiber samples collected on the lint filter, condenser and in the condensed water. Columns ‘Cotton’ and ‘Polyester’ contain the number of fibers of each type identified within the sample. These are used to calculate the percentage composition.

| **Cycle** | **Load** |  | **Cotton** | **Polyester** | **Cotton %** | **Polyester %** |  |
| --- | --- | --- | --- | --- | --- | --- | --- |
| **1** | **1** | Filter | 123 | 57 | 68.33 | 31.67 |  |
|  |  | Condenser | 165 | 97 | 62.98 | 37.02 |  |
|  |  | Water | 66 | 2 | 97.06 | 2.94 |  |
|  | **2** | Filter | 189 | 157 | 54.62 | 45.38 |  |
|  |  | Condenser | 192 | 70 | 73.28 | 26.72 |  |
|  |  | Water | 159 | 47 | 77.18 | 22.82 |  |
|  | **3** | Filter | 140 | 97 | 59.07 | 40.93 |  |
|  |  | Condenser | 127 | 84 | 60.19 | 39.81 |  |
|  |  | Water | 87 | 22 | 79.82 | 20.18 |  |
|  | **Mean** | **Filter** | **150.67** | **103.67** | **60.68** | **39.32** |  |
|  |  | **Condenser** | **161.33** | **83.67** | **65.48** | **34.52** |  |
|  |  | **Water** | **104.00** | **23.67** | **84.69** | **15.31** |  |
|  | **Std Dev** | Filter | 34.27 | 50.33 | 6.99 | 6.99 |  |
|  |  | Condenser | 32.65 | 13.50 | 6.90 | 6.90 |  |
|  |  | Water | 48.77 | 22.55 | 10.80 | 10.80 |  |
| **2** | **1** | Filter | 119 | 74 | 61.66 | 38.34 |  |
|  |  | Condenser | 99 | 41 | 70.71 | 29.29 |  |
|  |  | Water | 80 | 21 | 79.21 | 20.79 |  |
|  | **2** | Filter | 153 | 73 | 67.70 | 32.30 |  |
|  |  | Condenser | 131 | 83 | 61.21 | 38.79 |  |
|  |  | Water | 131 | 47 | 73.60 | 26.40 |  |
|  | **3** | Filter | 137 | 72 | 65.55 | 34.45 |  |
|  |  | Condenser | 136 | 66 | 67.33 | 32.67 |  |
|  |  | Water | 64 | 14 | 82.05 | 17.95 |  |
|  | **Mean** | Filter | **136.33** | **73.00** | **64.97** | **35.03** |  |
|  |  | Condenser | **122.00** | **63.33** | **66.42** | **33.58** |  |
|  |  | Water | **91.67** | **27.33** | **78.28** | **21.72** |  |
|  | **Std Dev** | Filter | 17.01 | 1.00 | 3.06 | 3.06 |  |
|  |  | Condenser | 20.07 | 21.13 | 4.81 | 4.81 |  |
|  |  | Water | 34.99 | 17.39 | 4.30 | 4.30 |  |
| **3** | **1** | Filter | 145 | 33 | 81.46 | 18.54 |  |
|  |  | Condenser | 117 | 30 | 79.59 | 20.41 |  |
|  |  | Water | 66 | 13 | 83.54 | 16.46 |  |
|  | **2** | Filter | 85 | 50 | 62.96 | 37.04 |  |
|  |  | Condenser | 180 | 134 | 57.32 | 42.68 |  |
|  |  | Water | 110 | 48 | 69.62 | 30.38 |  |
|  | **3** | Filter | 40 | 29 | 57.97 | 42.03 |  |
|  |  | Condenser | 105 | 47 | 69.08 | 30.92 |  |
|  |  | Water | 88 | 7 | 92.63 | 7.37 |  |
|  | **Mean** | Filter | **90.00** | **37.33** | **67.46** | **32.54** |  |
|  |  | Condenser | **134.00** | **70.33** | **68.67** | **31.33** |  |
|  |  | Water | **88.00** | **22.67** | **81.93** | **18.07** |  |
|  | **Std Dev** | Filter | 52.68 | 11.15 | 12.38 | 12.38 |  |
|  |  | Condenser | 40.29 | 55.79 | 11.14 | 11.14 |  |
|  |  | Water | 50.17 | 18.72 | 45.47 | 12.32 |  |
| **4** | **1** | Filter | 92 | 50 | 64.79 | 35.21 |  |
|  |  | Condenser | 114 | 48 | 70.37 | 29.63 |  |
|  |  | Water | 65 | 8 | 89.04 | 10.96 |  |
|  | **2** | Filter | 119 | 66 | 64.32 | 35.68 |  |
|  |  | Condenser | 109 | 7 | 93.97 | 6.03 |  |
|  |  | Water | 96 | 25 | 79.34 | 20.66 |  |
|  | **3** | Filter | 62 | 29 | 68.13 | 31.87 |  |
|  |  | Condenser | 94 | 36 | 72.31 | 27.69 |  |
|  |  | Water | 54 | 5 | 91.53 | 8.47 |  |
|  | **Mean** | Filter | **91.00** | **48.33** | **65.75** | **34.25** |  |
|  |  | Condenser | **105.67** | **30.33** | **78.88** | **21.12** |  |
|  |  | Water | **71.67** | **12.67** | **86.64** | **13.36** |  |
|  | **Std Dev** | Filter | 28.51 | 18.56 | 2.08 | 2.08 |  |
|  |  | Condenser | 10.41 | 21.08 | 13.10 | 13.10 |  |
|  |  | Water | 21.78 | 10.79 | 6.44 | 6.44 |  |
| **All** | **Mean** | Filter |  |  | **64.71** | **35.29** |  |
|  |  | Condenser |  |  | **69.86** | **30.14** |  |
|  |  | Water |  |  | **82.88** | **17.12** |  |
|  | **Std Dev** | Filter |  |  | 6.79 | 6.79 |  |
|  |  | Condenser |  |  | 9.88 | 9.88 |  |
|  |  | Water |  |  | 8.20 | 8.20 |  |
