# Supplementary material for: Impact of vented and condenser tumble dryers on waterborne and airborne microfiber pollution
Source: PLoS One. 2023 May 24;18(5):e0285548. doi: 10.1371/journal.pone.0285548 (PMC10208492; doi:10.1371/journal.pone.0285548)
Supplement: S3 Table — The table shows measured length (mm) and width (μm) of cotton and polyester fibers sampled from each of the three collection points at each of the four drying cycles. (DOCX) [file pone.0285548.s005.docx]

**S3 Table: Fiber length and width data for microfibers released from clean T-shirts in condenser tumble dryers.** The table shows measured length (mm) and width (μm) of cotton and polyester fibers sampled from each of the three collection points at each of the four drying cycles.

|  | **Lint Filter** | | | | **Condenser** | | | | **Water** | | | |
| --- | --- | --- | --- | --- | --- | --- | --- | --- | --- | --- | --- | --- |
|  | **Cotton** | | **Polyester** | | **Cotton** | | **Polyester** | | **Cotton** | | **Polyester** | |
|  | **Length (mm)** | **Width (µm)** | **Length (mm)** | **Width (µm)** | **Length (mm)** | **Width (µm)** | **Length (mm)** | **Width (µm)** | **Length (mm)** | **Width (µm)** | **Length (mm)** | **Width (µm)** |
| **Cycle 1** | 1.56 | 30.16 | 0.76 | 13.99 | 0.29 | 19.17 | 0.59 | 14.08 | 0.58 | 20.39 | 0.58 | 13.68 |
|  | 0.81 | 17.14 | 0.97 | 13.99 | 0.33 | 20.25 | 0.60 | 14.84 | 0.28 | 27.24 | 0.49 | 14.00 |
|  | 0.77 | 16.46 | 1.00 | 13.74 | 0.51 | 19.39 | 0.63 | 14.29 | 0.31 | 22.03 | 0.48 | 11.72 |
|  | 0.59 | 21.92 | 0.80 | 12.80 | 0.66 | 21.04 | 0.64 | 15.80 | 0.59 | 21.25 | 0.50 | 15.53 |
|  | 0.53 | 19.61 | 1.09 | 15.42 | 1.07 | 17.29 | 0.97 | 18.48 | 0.39 | 17.60 | 0.42 | 17.20 |
|  | 1.15 | 22.83 | 0.98 | 13.41 | 0.76 | 16.66 | 0.93 | 13.29 | 1.15 | 28.56 | 0.72 | 14.52 |
|  | 0.68 | 17.86 | 1.16 | 12.96 | 1.78 | 23.75 | 0.63 | 13.87 | 0.49 | 24.39 | 1.06 | 14.64 |
|  | 0.96 | 17.58 | 1.37 | 12.77 | 0.35 | 22.62 | 0.45 | 14.81 | 0.73 | 16.83 | 0.63 | 14.64 |
|  | 0.70 | 19.44 | 0.86 | 12.85 | 0.43 | 23.25 | 0.75 | 14.89 | 0.58 | 20.67 | 0.87 | 15.70 |
|  | 0.73 | 16.00 | 0.45 | 10.99 | 0.34 | 11.61 | 1.25 | 14.68 | 0.64 | 21.18 | 0.70 | 12.76 |
|  | 0.99 | 22.48 | 0.89 | 15.30 | 0.91 | 18.99 | 0.64 | 12.07 | 0.34 | 25.63 | 0.55 | 15.45 |
|  | 0.74 | 15.33 | 0.62 | 15.37 | 0.40 | 20.10 | 0.59 | 13.80 | 0.30 | 19.82 | 0.37 | 13.72 |
|  | 0.62 | 15.95 | 0.58 | 12.57 | 0.61 | 23.56 | 0.87 | 15.45 | 0.51 | 19.35 | 0.74 | 13.91 |
|  | 0.46 | 18.20 | 0.66 | 12.53 | 1.17 | 19.56 | 0.78 | 13.25 | 1.01 | 21.73 | 0.70 | 15.39 |
|  | 1.17 | 19.63 | 0.90 | 14.96 | 0.45 | 19.41 | 0.87 | 12.98 | 0.32 | 20.22 | 0.81 | 11.84 |
|  | 0.91 | 14.42 | 1.39 | 14.22 | 0.82 | 16.63 | 1.05 | 14.06 | 0.80 | 20.79 | 0.48 | 10.76 |
|  | 0.79 | 14.19 | 0.65 | 12.82 | 0.93 | 19.09 | 1.38 | 16.06 | 1.26 | 24.06 | 0.91 | 11.29 |
|  | 0.60 | 26.96 | 0.68 | 14.93 | 0.44 | 12.33 | 0.72 | 11.54 | 0.68 | 23.11 | 1.28 | 12.45 |
|  | 0.62 | 21.59 | 0.75 | 12.88 | 0.62 | 19.67 | 0.92 | 13.68 | 0.25 | 19.74 | 0.73 | 14.52 |
|  | 1.66 | 20.31 | 0.69 | 13.87 | 0.64 | 13.40 | 0.65 | 13.98 | 0.68 | 16.35 | 0.54 | 15.88 |
| **Cycle 1 Average** | **0.85** | **19.40** | **0.86** | **13.62** | **0.68** | **18.89** | **0.80** | **14.29** | **0.59** | **21.55** | **0.68** | **13.98** |
| **Cycle 1 Std Dev** | 0.32 | 4.10 | 0.25 | 1.18 | 0.37 | 3.45 | 0.24 | 1.50 | 0.29 | 3.21 | 0.23 | 1.72 |
| **Cycle 2** | 0.43 | 22.12 | 0.77 | 19.29 | 1.86 | 17.70 | 0.90 | 15.85 | 0.56 | 22.82 | 1.53 | 13.18 |
|  | 0.63 | 27.74 | 1.06 | 11.72 | 2.82 | 24.86 | 1.22 | 13.29 | 0.48 | 20.62 | 0.69 | 16.35 |
|  | 0.38 | 19.03 | 1.16 | 13.23 | 0.64 | 36.45 | 1.07 | 12.66 | 0.57 | 15.06 | 0.80 | 14.74 |
|  | 0.52 | 28.20 | 0.79 | 15.09 | 1.43 | 17.92 | 1.02 | 14.68 | 0.63 | 19.87 | 0.68 | 13.54 |
|  | 0.80 | 19.16 | 0.77 | 18.13 | 0.62 | 18.00 | 0.85 | 11.82 | 0.42 | 16.57 | 1.49 | 12.49 |
|  | 0.70 | 23.16 | 0.88 | 16.10 | 0.77 | 14.36 | 0.95 | 13.40 | 0.69 | 16.11 | 0.73 | 15.75 |
|  | 0.69 | 19.38 | 0.93 | 23.67 | 0.52 | 17.83 | 1.31 | 12.09 | 2.87 | 17.51 | 0.81 | 13.12 |
|  | 0.75 | 18.26 | 0.70 | 15.34 | 1.22 | 20.57 | 0.96 | 12.89 | 1.03 | 21.69 | 1.43 | 14.79 |
|  | 0.22 | 18.50 | 0.55 | 15.07 | 0.84 | 22.14 | 1.75 | 13.81 | 0.69 | 22.76 | 1.25 | 13.53 |
|  | 0.49 | 15.67 | 1.19 | 13.19 | 0.92 | 20.18 | 0.57 | 14.17 | 0.64 | 20.38 | 1.02 | 13.49 |
|  | 0.31 | 20.88 | 0.92 | 14.21 | 0.53 | 18.12 | 1.17 | 14.54 | 0.37 | 17.42 | 0.77 | 13.25 |
|  | 0.70 | 21.59 | 1.54 | 14.59 | 0.45 | 19.77 | 1.42 | 15.39 | 0.46 | 21.26 | 0.74 | 12.59 |
|  | 0.39 | 20.27 | 1.26 | 15.37 | 1.21 | 21.13 | 1.23 | 11.79 | 0.46 | 33.19 | 0.67 | 14.03 |
|  | 0.59 | 22.97 | 0.78 | 13.13 | 0.86 | 16.61 | 0.61 | 11.09 | 0.75 | 20.69 | 0.95 | 15.63 |
|  | 0.79 | 21.78 | 1.31 | 13.46 | 0.32 | 23.19 | 1.22 | 12.63 | 0.54 | 24.86 | 0.73 | 15.07 |
|  | 0.52 | 13.39 | 1.04 | 13.99 | 0.89 | 18.89 | 1.19 | 13.97 | 1.11 | 18.94 | 0.75 | 12.24 |
|  | 0.48 | 7.58 | 0.66 | 16.72 | 0.99 | 18.20 | 0.99 | 14.00 | 1.28 | 21.06 | 0.75 | 12.76 |
|  | 0.56 | 18.77 | 0.76 | 17.06 | 0.74 | 20.55 | 0.68 | 15.91 | 0.43 | 16.40 | 0.98 | 13.12 |
|  | 2.24 | 21.99 | 0.57 | 20.23 | 0.64 | 15.14 | 1.12 | 16.38 | 0.40 | 17.70 | 0.61 | 13.10 |
|  | 0.32 | 18.46 | 0.54 | 16.90 | 0.42 | 20.73 | 1.55 | 12.74 | 0.61 | 17.45 | 0.57 | 11.55 |
| **Cycle 2 Average** | **0.63** | **19.95** | **0.91** | **15.82** | **0.93** | **20.12** | **1.09** | **13.65** | **0.75** | **20.12** | **0.90** | **13.72** |
| **Cycle 2 Std Dev** | 0.42 | 4.52 | 0.28 | 2.85 | 0.58 | 4.61 | 0.30 | 1.49 | 0.56 | 4.04 | 0.30 | 1.28 |
| **Cycle 3** | 0.65 | 18.04 | 0.63 | 15.57 | 0.79 | 19.11 | 0.66 | 12.50 | 0.47 | 16.68 | 0.66 | 13.21 |
|  | 0.55 | 23.08 | 2.34 | 13.62 | 0.52 | 17.81 | 0.75 | 12.31 | 1.10 | 13.79 | 0.73 | 14.53 |
|  | 1.27 | 20.31 | 1.11 | 14.65 | 0.96 | 17.77 | 0.59 | 13.29 | 0.58 | 20.18 | 0.48 | 23.96 |
|  | 1.06 | 20.56 | 1.05 | 14.11 | 0.56 | 19.49 | 1.23 | 14.71 | 1.76 | 18.97 | 0.73 | 14.81 |
|  | 1.78 | 20.06 | 1.26 | 13.91 | 0.43 | 14.81 | 0.91 | 14.05 | 0.46 | 22.06 | 0.39 | 14.04 |
|  | 1.00 | 26.72 | 1.38 | 15.05 | 0.55 | 18.98 | 0.89 | 14.67 | 0.75 | 24.87 | 0.89 | 11.76 |
|  | 1.70 | 22.49 | 0.79 | 9.54 | 0.79 | 19.73 | 0.63 | 13.46 | 0.64 | 23.64 | 1.75 | 13.48 |
|  | 1.63 | 23.59 | 0.74 | 13.17 | 1.66 | 17.85 | 0.44 | 14.52 | 1.18 | 20.22 | 0.92 | 13.81 |
|  | 1.27 | 16.90 | 1.00 | 13.83 | 1.96 | 25.26 | 0.94 | 12.61 | 0.90 | 26.29 | 0.53 | 11.66 |
|  | 1.04 | 17.74 | 1.05 | 12.00 | 1.16 | 17.92 | 0.72 | 15.73 | 0.89 | 25.35 | 0.74 | 11.82 |
|  | 1.07 | 16.54 | 1.73 | 14.25 | 0.61 | 18.17 | 1.14 | 12.54 | 1.03 | 17.38 | 0.45 | 10.61 |
|  | 1.37 | 20.00 | 2.06 | 13.68 | 0.40 | 18.53 | 1.38 | 11.62 | 0.48 | 17.76 | 1.32 | 13.91 |
|  | 1.09 | 25.52 | 0.81 | 13.26 | 0.68 | 20.20 | 1.21 | 12.74 | 0.42 | 18.55 | 0.84 | 13.10 |
|  | 1.66 | 15.24 | 2.14 | 12.05 | 0.71 | 19.11 | 1.23 | 14.15 | 0.62 | 19.03 | 1.01 | 12.60 |
|  | 1.67 | 13.36 | 1.35 | 10.51 | 0.89 | 15.22 | 0.90 | 12.84 | 0.47 | 20.61 | 0.87 | 13.34 |
|  | 0.81 | 24.12 | 1.86 | 13.10 | 0.93 | 13.30 | 0.97 | 13.52 | 1.18 | 20.56 | 0.73 | 15.46 |
|  | 0.59 | 15.90 | 1.21 | 11.69 | 0.54 | 22.32 | 1.22 | 12.21 | 0.42 | 26.24 | 0.38 | 17.34 |
|  | 1.04 | 18.51 | 0.82 | 10.94 | 0.94 | 18.46 | 0.92 | 13.25 | 0.42 | 15.07 | 0.35 | 13.47 |
|  | 0.63 | 18.22 | 1.29 | 10.94 | 1.53 | 22.20 | 0.76 | 13.29 | 0.55 | 15.39 | 0.31 | 11.89 |
|  | 0.57 | 30.24 | 1.20 | 11.39 | 0.42 | 17.38 | 0.74 | 9.96 | 0.63 | 20.35 | 0.72 | 12.70 |
| **Cycle 3 Average** | **1.12** | **20.36** | **1.29** | **12.86** | **0.85** | **18.68** | **0.91** | **13.20** | **0.75** | **20.15** | **0.74** | **13.88** |
| **Cycle 3 Std Dev** | 0.41 | 4.25 | 0.49 | 1.64 | 0.43 | 2.66 | 0.26 | 1.27 | 0.35 | 3.70 | 0.35 | 2.81 |
| **Cycle 4** | 1.16 | 19.87 | 1.02 | 11.58 | 1.53 | 21.09 | 0.96 | 13.75 | 0.68 | 18.55 | 0.77 | 13.68 |
|  | 1.33 | 22.97 | 0.79 | 12.79 | 1.35 | 18.31 | 0.62 | 15.50 | 0.63 | 18.61 | 0.56 | 11.99 |
|  | 0.87 | 18..514 | 0.83 | 11.58 | 0.56 | 20.59 | 1.57 | 12.96 | 0.64 | 16.67 | 0.77 | 11.37 |
|  | 0.77 | 25.89 | 1.13 | 12.30 | 1.22 | 21.58 | 0.95 | 12.66 | 0.56 | 14.54 | 0.47 | 13.35 |
|  | 1.48 | 24.76 | 2.03 | 13.23 | 0.98 | 18.07 | 1.20 | 11.99 | 0.69 | 21.30 | 0.67 | 12.15 |
|  | 0.93 | 20.63 | 1.01 | 13.51 | 0.46 | 23.32 | 1.74 | 11.65 | 1.66 | 13.35 | 0.61 | 15.42 |
|  | 0.52 | 19.65 | 1.07 | 14.92 | 0.39 | 16.36 | 1.66 | 12.78 | 1.02 | 11.59 | 0.63 | 13.77 |
|  | 1.22 | 13.38 | 1.77 | 14.09 | 0.71 | 19.52 | 0.71 | 12.15 | 2.14 | 17.24 | 0.45 | 9.26 |
|  | 1.71 | 20.11 | 0.65 | 13.38 | 0.53 | 20.90 | 1.16 | 11.45 | 2.24 | 20.33 | 0.52 | 13.77 |
|  | 1.65 | 18.52 | 0.86 | 11.13 | 0.99 | 25.87 | 1.06 | 11.02 | 0.72 | 17.72 | 0.71 | 12.90 |
|  | 1.70 | 16.73 | 0.71 | 11.61 | 1.25 | 18.73 | 0.90 | 15.60 | 0.58 | 16.27 | 0.58 | 17.30 |
|  | 0.96 | 23.08 | 0.95 | 11.37 | 1.15 | 18.46 | 0.79 | 14.24 | 0.39 | 18.98 | 0.80 | 11.92 |
|  | 0.73 | 14.30 | 0.82 | 10.67 | 0.71 | 19.60 | 1.21 | 12.48 | 0.47 | 16.32 | 0.59 | 9.82 |
|  | 1.87 | 20.33 | 1.06 | 11.22 | 0.44 | 16.49 | 0.94 | 14.47 | 0.29 | 15.22 | 0.53 | 12.53 |
|  | 0.50 | 22.42 | 1.10 | 11.64 | 0.90 | 14.81 | 0.97 | 14.50 | 0.79 | 16.66 | 0.46 | 11.22 |
|  | 0.78 | 14.30 | 0.91 | 15.63 | 0.74 | 21.19 | 1.01 | 14.00 | 0.35 | 13.97 | 0.61 | 12.75 |
|  | 2.47 | 14.85 | 0.99 | 15.32 | 0.86 | 20.14 | 0.62 | 16.06 | 0.46 | 19.66 | 0.43 | 17.94 |
|  | 1.95 | 15.50 | 0.89 | 16.13 | 0.34 | 15.49 | 0.78 | 12.93 | 0.51 | 15.67 | 0.85 | 12.16 |
|  | 1.13 | 13.64 | 0.61 | 15.68 | 0.67 | 24.82 | 1.23 | 14.47 | 0.22 | 19.49 | 0.51 | 12.19 |
|  | 1.63 | 15.86 | 0.74 | 15.75 | 0.62 | 19.36 | 0.62 | 16.49 | 0.22 | 19.29 | 0.56 | 13.05 |
| **Cycle 4 Average** | **1.27** | **18.78** | **1.00** | **13.18** | **0.82** | **19.74** | **1.04** | **13.56** | **0.76** | **17.07** | **0.60** | **12.93** |
| **Cycle 4 Std Dev** | 0.53 | 3.94 | 0.35 | 1.85 | 0.34 | 2.88 | 0.33 | 1.60 | 0.58 | 2.53 | 0.12 | 2.11 |
| **Overall Average** | **0.97** | **19.63** | **1.01** | **13.87** | **0.82** | **19.36** | **0.96** | **13.68** | **0.71** | **19.72** | **0.73** | **13.62** |
| **Overall Std Dev** | **0.49** | **4.17** | **0.39** | **2.26** | **0.44** | **3.47** | **0.30** | **1.50** | **0.46** | **3.73** | **0.28** | **2.06** |
