# Supplementary material for: Impact of vented and condenser tumble dryers on waterborne and airborne microfiber pollution
Source: PLoS One. 2023 May 24;18(5):e0285548. doi: 10.1371/journal.pone.0285548 (PMC10208492; doi:10.1371/journal.pone.0285548)
Supplement: S4 Table — The table shows measured mass of the wash load used (kg) and microfibers collected (mg) for on the dryer lint filter, on the condenser and in the condensed water for the condenser dryer and on the dryer lint filter and from the exhaust vent for the vented dryer. These data are used to calculate quantity of microfibers at these stages in terms of ppm (parts per million, i.e., mg microfiber released per kg dry wash load) and percentage lint filter efficiency for each of the four drying cycles. (DOCX) [file pone.0285548.s006.docx]

**S4 Table. Gravimetric quantification of microfiber release from real consumer loads in condenser and vented tumble dryers.** The table shows measured mass of the wash load used (kg) and microfibers collected (mg) for on the dryer lint filter, on the condenser and in the condensed water for the condenser dryer and on the dryer lint filter and from the exhaust vent for the vented dryer. These data are used to calculate quantity of microfibers at these stages in terms of ppm (parts per million, i.e. mg microfiber released per kg dry wash load) and percentage lint filter efficiency for each of the four drying cycles.

|  | **Load** | **Load mass (kg)** | **Microfiber mass**  **(mg)** | | | | **Microfiber release**  **(ppm)** | | | | | **Lint filter efficiency**  **(%)** |
| --- | --- | --- | --- | --- | --- | --- | --- | --- | --- | --- | --- | --- |
|  |  |  | **Lint Filter** | **Condenser** | | **Water** | **Lint Filter** | **Condenser** | | **Water** | **Total** |  |
| **Condenser** | 1 | 3.57 | 888.37 | 38.95 | | 16.45 | 248.84 | 10.91 | | 4.61 | 264.36 | 94.13 |
|  | 2 | 2.03 | 366.07 | 41.89 | | 15.17 | 180.33 | 20.64 | | 7.47 | 208.44 | 86.52 |
|  | 3 | 1.88 | 785.96 | 48.76 | | 14.53 | 418.06 | 25.93 | | 7.73 | 451.73 | 92.55 |
|  | 4 | 2.97 | 1347.19 | 68.90 | | 18.03 | 453.60 | 23.20 | | 6.07 | 482.87 | 93.94 |
|  | 5 | 3.59 | 908.55 | 71.67 | | 20.42 | 253.08 | 19.96 | | 5.69 | 278.73 | 90.80 |
|  | 6 | 2.85 | 840.57 | 43.98 | | 11.01 | 294.94 | 15.43 | | 3.86 | 314.23 | 93.86 |
|  | 7 | 1.86 | 331.27 | 40.90 | | 15.71 | 178.10 | 21.99 | | 8.44 | 208.53 | 85.41 |
|  | 8 | 3.16 | 1513.66 | 109.56 | | 30.12 | 479.01 | 34.67 | | 9.53 | 523.21 | 91.55 |
|  | **Mean** | **2.74** | **872.71** | **58.07** | | **17.68** | **313.25** | **21.59** | | **6.68** | **341.51** | **91.09** |
|  | **Std Dev** | **0.72** | **413.33** | **24.38** | | **5.71** | **120.83** | **7.05** | | **1.95** | **126.02** | **3.39** |
|  | **Load** | **Load mass (kg)** | **Microfiber mass**  **(mg)** | | | | **Microfiber release**  **(ppm)** | | | | | **Lint filter efficiency**  **(%)** |
|  |  |  | **Lint Filter** | | **Vent** | | **Lint Filter** | | **Vent** | | **Total** |  |
| **Vented** | 1 | 1.57 | 402.36 | | 70.66 | | 256.28 | | 45.01 | | 301.29 | 85.06 |
|  | 2 | 4.64 | 1679.05 | | 73.96 | | 361.87 | | 15.94 | | 377.81 | 95.78 |
|  | 3 | 3.74 | 355.22 | | 89.78 | | 94.98 | | 24.01 | | 118.98 | 79.82 |
|  | 4 | 2.00 | 464.18 | | 87.35 | | 232.09 | | 43.67 | | 275.76 | 84.16 |
|  | 5 | 2.98 | 589.31 | | 51.65 | | 197.76 | | 17.33 | | 215.09 | 91.94 |
|  | 6 | 2.16 | 416.36 | | 82.14 | | 192.76 | | 38.03 | | 230.79 | 83.52 |
|  | 7 | 3.04 | 711.26 | | 98.65 | | 233.97 | | 32.45 | | 266.42 | 87.82 |
|  | 8 | 1.88 | 398.15 | | 93.79 | | 211.78 | | 49.89 | | 261.67 | 80.93 |
|  | **Mean** | **2.75** | **626.99** | | **81.00** | | **222.69** | | **33.29** | | **255.98** | **86.13** |
|  | **Std Dev** | **1.05** | **441.25** | | **15.17** | | **74.32** | | **13.01** | | **74.16** | **5.46** |

***Microfiber release (ppm) = Microfiber mass (mg) / Load mass (kg)**

**^†^Lint filter efficiency is the percentage of total microfiber release collected on the lint filter**
