# Supplementary material for: Impact of vented and condenser tumble dryers on waterborne and airborne microfiber pollution
Source: PLoS One. 2023 May 24;18(5):e0285548. doi: 10.1371/journal.pone.0285548 (PMC10208492; doi:10.1371/journal.pone.0285548)
Supplement: S5 Table — The table shows details of representative examples of tumble dryers sold under 24 different brands in the UK market, with details of instructions regarding cleaning of lint filter. (DOCX) [file pone.0285548.s007.docx]

**S5 Table: Survey of lint filter cleaning instructions in tumble dryers.** The table shows details of representative examples of tumble dryers sold under 24 different brands in the UK market, with details of instructions regarding cleaning of lint filter.

| **Brand** | **Parent** | **Model** | **Type** | **Guidance** |
| --- | --- | --- | --- | --- |
| **Indesit** | Whirlpool | I2D81WUK | Condenser | **Clean under running water or use vacuum cleaner** |
| **Hotpoint** | Whirlpool | H1D80WUK | Vented | **Clean under running water or use vacuum cleaner** |
| **Candy** | Haier | CSEC9DF | Condenser | Use a soft brush or fingertips |
| **Hoover** | Haier | HLEV10LG | Vented | Use a soft brush or fingertips |
| **Fisher & Paykel** | Haier | DH9060P2 | Heat Pump | Use hand or soft cloth |
| **Baumatic** | Haier | BBTDH7A1TE | Heat Pump | Use a soft brush or fingertips |
| **Haier** | Haier | HD90-A2979S | Heat Pump | **No specific instructions** |
| **Beko** | Arcelik | DTLCE80021W | Condenser | Use hands or soft cloth |
| **Grundig** | Arcelik | GT76824EW | Heat Pump | Use hands, brush or vacuum cleaner |
| **Siemens** | BSH | WQ45G209GB | Heat Pump | **Hand followed by washing under water** |
| **Neff** | BSH | R8580X3GB | Condenser | **Rinse fluff under running water or use a dishwasher** |
| **Bosch** | BSH | WTN83201GB | Condenser | **Rinse fluff under running water or use a dishwasher** |
| **AEG** | Electrolux | T7DBG840N | Heat Pump | Use hands or vacuum cleaner |
| **Zanussi** | Electrolux | ZTE7101PZ | Vented | Use a moist hand |
| **Miele** | Miele | TWF760WP | Heat Pump | Use hands or a vacuum cleaner |
| **Samsung** | Samsung | DV80TA020AE | Heat Pump | Use brush provided |
| **LG** | LG | FDV909S | Heat Pump | **No specific instructions** |
| **Electra** | ECP | TDC7100B | Condenser | Use hands or soft cloth |
| **Montpellier** | Montpellier | MIHP75 | Heat Pump | Use soft brush or fingertips |
| **Hisense** | Hisense | DCGE802B | Condenser | Use hands |
| **Sharp** | Sharp / Vestel | KD-NCB8S7GB9 | Condenser | Hands or soft cloth |
| **Logik (Currys)** | Store brand | LVD7W18 | Vented | Use a cloth |
| **John Lewis (JLP)** | Store brand | JLTDH25 | Heat Pump | Use your hand. **Avoid use of water to protect environment** |
| **Bush (Argos)** | Store brand | TD7CDBCW | Condenser | Use hands or soft cloth |
