# Supplementary material for: Impact of vented and condenser tumble dryers on waterborne and airborne microfiber pollution
Source: PLoS One. 2023 May 24;18(5):e0285548. doi: 10.1371/journal.pone.0285548 (PMC10208492; doi:10.1371/journal.pone.0285548)
Supplement: Attachment — Submitted filename: Response to Reviewers.docx [file pone.0285548.s008.docx]

20^th^ March 2023

Dear Dr Amitava Mukherjee,

**Re: Impact of vented and condenser tumble dryers on waterborne and airborne microfiber pollution (PONE-D-23-02944)**

Thank you for considering the above manuscript for publication in PLOS ONE. Attached is a revised version of the manuscript which I hope reflects the revisions requested in your letter dated 9^th^ March 2023.

**Journal requirements:**

**Point 1**: I confirm that the manuscript meets PLOS ONE’s style requirements, including those for file naming.

**Points 2 and 3**: Regarding funding, we have removed mention of any funding information from the Acknowledgements or elsewhere in the manuscript, as requested. Furthermore, we have confirmed that the Worshipful Company of Launderers does not provide grant numbers with their awards. Below is a revised Funding Statement to reflect this.

The study was entirely funded by the following three sources:

The Worshipful Company of Launderers provided a grant to AMC through the Master (2022) of that institution and its Education Committee. No grant number was provided. The Worshipful Company of Launderers had no role in study design, data collection and analysis, decision to publish, or preparation of the manuscript.

<https://www.launderers.co.uk/>

Northumbria University funded the study through employment of JRD and SL, and provision of consumables. Only the co-authors affiliated to this institution were involved in study design, data collection and analysis, decision to publish, and preparation of the manuscript.

<https://www.northumbria.ac.uk/>

Procter & Gamble Technical Centres Ltd provided funding in the form of salaries for NJL, AKM and AJS and purchase of appliances and related laboratory consumables. In addition to NJL, AKM and AJS, another member of Procter & Gamble staff contributed to the study as described in the acknowledgements but only these individuals were involved in study design, data collection and analysis, decision to publish, and preparation of the manuscript. Procter & Gamble management gave approval to publish, but this process did not influence the text of the manuscript.

<https://www.pg.com/>

**Point 4:** Details regarding ethical approval for the study have been added to the manuscript at line 182.

**Point 5:** The reference list is complete and correct, and one reference has been added at the request of a reviewer (see below) compared to the original submission.

**Reviewers’ comments**

**Abstract:** This has been edited to sharpen the narrative and reduce its size to 240 words which is well below the recommended limit of 300 words.

**Introduction**

**Line 85-86:** A reference has been included citing the legislation discussed.

The reviewer comments that “the requirements of comparing vented and condenser dryers was not clear. How this previously studied Tumble dryers are different from the vented and condenser tumble dryers”. We believe that this point is addressed in the Abstract and Introduction section “Contribution of tumble drying” where the previous work – which has all been focused on vented dryers, i.e. not involving condenser dryers, is summarized and the novelty of the present work clearly articulated in being the first to study condenser dryers and the first to study real consumer wash loads rather than new, clean, fabrics.

**Line 130-145:** The reviewer commented that “existing both vented, and condenser are designed to store their filtered fibers in it. However, their cleaning process and methods are dependent on the user decision. Based on these pitfalls, study has taken two different objectives that did not address the issue discussed in line 130-145. These issues can be included as an aim of the study”.

I am interpreting this as a comment that the article contains measurements and discussion regarding disposal of fibers collected on the lint filter, which could result in waterborne pollution if consumers clean these components using water, but this is not set out a as a specific aim. The Introduction has been updated to explicitly build this into the aims.

**Materials and methods**

**Line 195**: The comment relates to whether common standards, e.g. ISO or BS, are relevant to the protocols. There are no such standards available for this type of research, although the methods employed have been successfully used in our previous articles (e.g. the two Lant *et al.* references) and articles cited in those.

**Line 264**: As described above (line 195) there is no AATCC method or other standard for this methodology.

**Results and discussion**

We found the general comments relating to this section rather confusing, especially the comment that “the study cited the findings of the previous literatures that evaluated the normal tumble dryers emission, this study did not evaluate it, to compare with the considered to varieties”. The present study comprehensively reviews and acknowledges the previous research in this area of microfiber pollution from tumble dryers. Those studies relate to airborne pollution from vented dryers. As condenser dryers are far more commercially important in other markets such as Europe where vented dryers are being phased out, this is the first study relating to condenser dryers, the first study to recognize that condenser dryers are a source of microfiber pollution from three sources, and a clear comparison is made versus vented dryers using consumer wash loads and the same test methodology. This novelty versus the earlier work of Kapp and Miller and others is clearly articulated in the abstract and introduction. It is not a case of ‘new designs’ (assumed to be condenser dryers) and ‘old designs’ (assumed to be vented dryers). Both types of dryer have been sold concurrently on the market for many years. As we have articulated, vented dryers cause airborne microfiber pollution (as described in earlier works) and may cause waterborne pollution depending on lint filter disposal method (present study) whereas condenser dryers have three potential sources of waterborne pollution – condensed water, cleaning of condenser, and cleaning of lint filter (if consumers wash this under water). The reviewer suggests that we should be including discussion of the relative superiority of ‘new’ (assumed to be condenser) versus ‘old’ (assumed to be vented) designs. We respectfully suggest that this is outside the scope of the study as that would involve weighing up the relative impact of causing air pollution (associated with vented dryers) or water pollution (mainly associated with condenser dryers). Our hope is that this article will bring the attention of the scientific community to the contribution of condenser dryers to microfiber pollution for the first time with an expectation that other groups may conduct further studies to either mitigate the issues or better understand the environmental impacts.

The reviewer also commented that there is no comparison with the microfiber release at the washing stage included in the present study to better understand the relative impact of washing and drying on microfiber pollution. We believe that this point is already addressed by Fig 8 which uses results from our previous comprehensive study of down-the-drain microfiber release from real consumer loads in washing machines (using similar appliances and washing conditions to those used in the present study) to make a holistic calculation of total microfiber release from all points during the washing and drying process. It is very clear from this data that when consumers dispose of dryer lint in municipal solid waste, that the washing stage will release more fibers to the environment than the tumble drying stage, although quantities released during drying are still very significant. However, if consumers follow the instructions of some dryer manufacturers and clean dryer lint filters in water, tumble drying becomes a more significant contributor than the washing stage.

The reviewer’s final point is that disposal of collected microfibers in municipal solid waste will directly contribute to terrestrial microfiber pollution. This is an interesting argument and one that deserves further debate given that the main solution being implemented to mitigate microfiber pollution during laundering involves installation of filtration systems that involve disposal of the residue in municipal solid waste. We have updated the manuscript at the end of the Conclusions to highlight this point that disposal of microfibers in municipal solid waste could lead to some further environmental impacts that deserve further study.

We look forward to hearing from you.

Yours sincerely,

Dr Neil Lant, on behalf of the authors

lant.n@pg.com

R&D Senior Director / Research Fellow, Procter & Gamble
