# Supplementary material for: Impact of vented and condenser tumble dryers on waterborne and airborne microfiber pollution
Source: PLoS One. 2023 May 24;18(5):e0285548. doi: 10.1371/journal.pone.0285548 (PMC10208492; doi:10.1371/journal.pone.0285548)
Supplement: Attachment — Submitted filename: Response to Reviewers.docx [file pone.0285548.s009.docx]

18^th^ April 2023

Dear Dr Amitava Mukherjee,

**Re: Impact of vented and condenser tumble dryers on waterborne and airborne microfiber pollution (PONE-D-23-02944)**

Thank you for considering our revised manuscript. Attached is a second revision of the manuscript reflecting the amendments requested in your letter dated 12^th^ April 2023.

**Journal requirements:**

I confirm that the manuscript meets PLOS ONE’s style requirements and that no further changes have been made to the reference list.

**Reviewers’ comments**

I thank Reviewer #1 for their comment that AATCC has released a standard method for the quantification of microfiber release. This is welcome progress, and I was involved in the early stages of its development. However, as it is an accelerated method, and limited in scope to microfiber release during clothes washing rather than drying, it was not suitable for the present study.

Reviewer #2 requested two minor revisions to lines 247-248 and line 498 which have been made. The first is an important correction and we agree that the latter is a useful clarification.

Reviewer #3 commented that the abstract contains no data was included in the abstract to support the comments “high levels of microfibers” (line 33) and “vented dryers were found to be significant contributors” (line 35). The abstract has been updated to include all of these data.

Reviewer #3 asked about precautions to reduce airborne microfiber contamination. In addition to general good laboratory practice including wearing of laboratory coats, several references to additional precautions are mentioned in the manuscript including use of orange cotton and black polyester garments in line 182 (in order to easily differentiate these, and enable easy identification of any contaminant fibers), appliance cleanout procedures (lines 204 and 215) and use of a specialized laboratory with high levels of ventilation (line 230).

Reviewer #3 also asked that we include details of statistical software in the Statistics section (line 311-315). This has been updated accordingly.

We hope that these revisions are satisfactory and sufficient to enable acceptance.

Yours sincerely,

Dr Neil Lant, on behalf of the authors

lant.n@pg.com

R&D Senior Director / Research Fellow, Procter & Gamble
